# Did Laos really control the transmission of SARS-CoV-2 in 2020?

**DOI:** 10.1016/j.lanwpc.2021.100202

**Published:** 2021-07-20

**Authors:** Barnaby Flower, Michael Marks

**Affiliations:** 1 Oxford University Clinical Research Unit, Ho Chi Minh City, Vietnam; 2 Clinical Research Department, Faculty of Infectious and Tropical Diseases, London School of Hygiene & Tropical Medicine, London; 3 Hospital for Tropical Diseases, London, United Kingdom

One of the most compelling aspects of the COVID-19 pandemic has been its uneven global impact. Once the scale of the COVID-19 epidemic in Wuhan became clear, large outbreaks were expected to follow in neighboring Asian states [1]. However, due to a combination of effective public health interventions, transmission of SARS-CoV-2 was largely suppressed. By the end of September 2020, just over 500 COVID-19 related deaths had been reported across mainland South East Asia, while hundreds of thousands were dying in Europe and the Americas.

Lao Peoples Democratic Republic (Lao PDR) has limited public health infrastructure but reported a particularly low burden of disease. Despite bordering China, it was the last country in South East Asia to report a confirmed case of COVID-19. The government implemented a national lockdown soon after and closed all land borders. By September 2020, months after schools and travel networks had reopened, only 23 cases had been identified. The first COVID-19–related death was not reported until 9^th^ May 2021.

In low resource settings, the accuracy of routinely reported case numbers and deaths may be difficult to verify. For example, Peru more than doubled its official COVID-19 death toll due to an under-count attributed to a lack of testing [2]. Lao PDR's reported case numbers are based on the swabbing of symptomatic individuals and foreign arrivals, which could result in under-reporting of minimally or asymptomatic cases in the community. Alternative methods of surveillance, such as sero-surveys are therefore essential tools for monitoring the pandemic.

To address whether SARS-CoV-2 may have circulated more widely in the first months of the pandemic, Virachith and colleagues conducted an extensive SARS-CoV-2 antibody seroprevalence survey in Lao PDR in August and September 2020 [3]. They evaluated antibody prevalence in three important populations: members of the general community, healthcare workers and individuals who work closely with bats and rural wildlife in Vientiene province. The latter in particular, represents a population of considerable interest, given that bats and rodents in the region are frequently infected with a diverse array of alpha- and beta- coronaviruses, including SARS-CoV-2–like viruses [4].

The study is the largest SARS-CoV-2 antibody survey in South East Asia to date and provides the first estimation of community seroprevalence in the region. They found only 0.1% individuals surveyed had antibodies to both SARS-CoV-2 nucleoprotein and spike protein. Both individuals tested negative by lateral flow immunoassay and probably represent false positives. By comparison, all samples from COVID-19-cases identified by national COVID-19 surveillance in Lao PDR (n=15) were seropositive by both ELISA and rapid diagnostic tests. Given the breadth of the study, the data strongly supports the hypothesis that there was minimal SARS-Co-V-2 circulation in Lao-PDR up to September 2020.

The study also found that antibodies against nucleoprotein were more prevalent in villagers employed in harvesting bat guano (20%) than in the general population (5%) or health care workers (2%). Nucleoprotein is more conserved across coronaviruses than spike protein and this finding likely represents cross-reactivity with non-SARSCoV2 coronaviruses. Data from other settings suggest these cross-reactive antibodies do not offer protection from COVID-19 [5]. Although nucleoprotein antibody prevalence in Lao PDR probably has no relevance to its successful suppression of COVID-19, the elevated prevalence in bat guano collectors does imply that specific rural populations have significant exposure to coronaviruses, representing an ongoing risk for emergence of future pandemics.

A major strength of this study is the sampling strategy. Previous surveys have been compromised by selection bias, including non-random, unrepresentative sampling [6], substantial dropout [7], or convenience sampling [8], Virachith et al use a multistage cluster sampling design with random selection of districts within five separate provinces and report minimal voluntary drop out. Some bias may have arisen from ‘unavailable’ individuals being replaced by other randomized participants, who may have a lower risk of exposure to COVID-19 by virtue of not being at work or school. Overall, however, the randomized nature of the sampling and the very low antibody prevalence limits the impact of this bias.

The findings are in keeping with data from Lao PDR's South East Asian neighbours [9], [10], [11]. Taken together this data illustrates that there was no widespread silent transmission of SARS-CoV-2 in South East Asia in 2020 and re-affirms that these countries’ success in limiting deaths from COVID-19 is due to effective suppression of SARS-CoV-2 transmission, through timely public health interventions. However, for Lao PDR and its neighbors the battle is far from over. Since April 2021 there has been sustained community transmission of SARS-CoV-2 throughout South East Asia even in countries which had successfully suppressed transmission throughout 2020. Vaccination coverage remains woefully inequitable with almost all doses administered in a small number of high-income countries. As high vaccination coverage is likely the only sustainable strategy to suppress SARS-CoV-2 transmission in the long term and reduce the ongoing risk of new variants emerging addressing this vaccination-gap must be a priority.
